# Near infrared fluorophore specific for annexin A2 identifies peripheral nerve injury in a rodent transection model

**DOI:** 10.3389/fcell.2026.1657209

**Published:** 2026-04-29

**Authors:** Marie C. Spezia, Christopher J. Dy, Brooke Farrell, Ayan Iftikhar, Stephen DeMartini, Ron Perez, Samuel Achilefu, David M. Brogan

**Affiliations:** 1 University of Missouri School of Medicine, Columbia, MO, United States; 2 Department of Orthopaedic Surgery, Washington University School of Medicine, St. Louis, MO, United States; 3 Washington University, MO, United States; 4 Department of Biomedical Engineering, University of Texas Southwestern Medical Center, Dallas, TX, United States

**Keywords:** near infrared fluorophore, nerve imaging, nerve injury, peripheral nerve repair, Wallerian degeneration

## Abstract

**Introduction:**

Peripheral nerve injuries (PNI) pose a major challenge in the intraoperative assessment of nerve viability. Wallerian degeneration (WD) is the degenerative process of axonal breakdown distal to the site of injury, with limited proximal extension, and progresses through spatiotemporally consistent stages facilitated by Schwann cell reprogramming and macrophage infiltration. We evaluated LS-301, a near-infrared (NIR) fluorophore previously used in human oncology trials, that targets phosphorylated annexin A2 (pANXA2), which is upregulated and redistributed to Schwann cell surfaces after nerve injury.

**Methods:**

Utilizing a sciatic nerve transection model in rats, LS-301 was compared with the untargeted control dye Cypate-3 using in vivo fluorescence imaging. Fluorescence intensities in proximal and distal bulbs and their adjacent segments were quantified, normalized to the contralateral sham nerve, and compared across time points chosen to represent documented stages of WD (5 h, 2 days, and 2 weeks).

**Results:**

LS-301 showed time-dependent accumulation in injured nerve in a spatial pattern consistent with the expected progression of WD, whereas Cypate-3 uptake was variable and lacked a consistent spatiotemporal gradient.

**Discussion:**

This study’s findings support the feasibility of using a targeted NIR probe to preferentially label degenerating versus uninjured peripheral nerve after transection, with potential for intra-operative application. This proof-of-concept study does not define per-nerve surgical decision thresholds yet, but this will be the focus of future work testing whether LS-301 mapping can complement existing intraoperative assessments and determine surgically actionable fluorescence cutoffs.

## Introduction

Peripheral nerve injuries (PNIs) are common sequelae of trauma and iatrogenic injuries and occur with a mean national incidence of around 11 events per 100,000 population per year in the United States (Zhang et al., 2022; Hussain et al., 2020; Xu et al., 2021; Zhang M. et al., 2021; Murphy et al., 2023). The degree of axonal injury and predicted prognosis following nerve repair can be difficult to ascertain intraoperatively. In the context of full nerve transection specifically, the extent of Wallerian degeneration (WD) present in the distal stump and, to a lesser extent in the proximal stump, are key points that could aid in decision making but that cannot be appreciated with intraoperative inspection. This information has the potential to dramatically change surgical management, which could range from expectant management to resection of damaged nerve and grafting. Despite the myriad of advancements in nerve repair, an intraoperative technique of assessing degree of nerve injury in terms of area involved in degeneration, a fact that correlates with prognosis, has remained frustratingly elusive (Wilson et al., 2022; Park et al., 2025).

As it stands, surgeons often rely on gross appearance of the nerve including color and texture with the additional assistance of electrical stimulation or intraoperative nerve conduction testing when these modalities are feasible. Still, even in the most experienced hands, these techniques can be imprecise and rudimentary. For example, immediately after injury, distal axons may continue to conduct electrical signals until Wallerian degeneration evolves while, conversely, chronically denervated axons have elevated thresholds and reduced responses, risking false negatives or overestimation of lesion severity intraoperatively (Pripotnev et al., 2022; Wu et al., 2020; Zelenski et al., 2023). Additionally, a measurement indicating disrupted conduction across a segment cannot discern the extent of injury nor can it provide information as to the state of the inflammatory microenvironment including Schwann cell injury/repair programs (Pripotnev et al., 2022; Zelenski et al., 2023). Targeted molecular imaging could bridge this gap by labeling Wallerian degeneration-specific molecular changes, but previous attempts to accomplish this with a variety of probes have yet to find consistent success (Wilson et al., 2022; Hingorani et al., 2018; Wang and Gibbs, 2023; Wang et al., 2020; Holland, 2002). Wallerian degeneration is the canonical process that encompasses the gradient of metabolic and structural changes that occur in a peripheral nerve over the days to weeks following axonal injury (Zhang K. et al., 2021; Eder et al., 2017). In addition to biochemical cascades and cytoskeletal alterations that unfold within the axon itself, this mechanism of nerve injury response also relies on the recruitment of proinflammatory immune cell migration and the transdifferentiation of Schwann cells from myelinating to a pro-regenerative phenotype (Yi et al., 2017; Boissonnas et al., 2020; Nocera and Jacob, 2020). Uniquely, these alterations in the injured nerve and its environment display gradation both spatially and temporally relative to the zone of injury (Zhao et al., 2022; Seitz et al., 1989; Rotshenker, 2011).

The importance of axonal continuity with its cell body is underscored by the effects observed in the WldS mouse phenotype. The WldS mutation is a gain-of-function mutation that results in increased nicotinamide mononucleotide adenylyltransferase (NMNAT) activity (Yahata et al., 2009; Sasaki et al., 2009a; Avery et al., 2009). This protein results from a chimeric fusion of the N-terminal portion of Ube4b and full-length Nmnat1, and the NMNAT enzymatic activity is essential for its neuroprotective effects as increased NMNAT activity contributes to prolonged NAD + availability in the segment of the axon distal to the site of injury causing disrupted continuity from the cell body (Sasaki et al., 2009a; Avery et al., 2009; Sasaki et al., 2009b; Wang et al., 2015). Transcriptional profiling of injured sciatic nerves in WldS mice showed significant delays in axonal regeneration compared to wild-type mice (Avery et al., 2012). WldS preserves axons but also delays the production of signals essential for the nerve repair process.

Recent research has identified annexin A2 (ANXA2) as a potential molecular target related to nerve injury response (Sasaki et al., 2009b; Wang et al., 2015). Annexin A2 is a calcium-dependent phospholipid-binding protein that, in its active form, often exists as a heterotetramer complex comprised of two annexin A2 monomers bound to an S100A10 dimer and has been shown to be involved in processes of membrane repair, fibrinolysis, and inflammation (Dallacasagrande and Hajjar, 2020; Yamanaka et al., 2016; Liu et al., 2019; Hayashi et al., 2007). Annexin A2 is present in peripheral nerves at baseline. This protein is synthesized within the nerve but remains isolated in Schwann cell Schmidt–Lanterman incisures and paranodal loops in a healthy nerve whereas ANXA2 is phosphorylated and translocated to the cell surface in an injury setting (Hayashi et al., 2007). After an insult, signals such as the hydrogen peroxide released from damaged axons drive changes in Schwann cells that include redistribution of ANXA2 and cytoskeletal remodeling, promoting an orientation towards repair functions (Nocera and Jacob, 2020; Hayashi et al., 2007; Negro et al., 2018). ANXA2 has been shown to undergo post-translational phosphorylation at tyrosine 23 which directs the translocation of phosphorylated ANXA2 (pANXA2) complex to the cell surface in Schwann cells where it acts as a receptor for tissue plasminogen activator (tPA) and plasminogen, greatly enhancing local plasmin generation particularly at the site of injury (Yamanaka et al., 2016; Liu et al., 2019; Negro et al., 2018; Koerdt and Gerke, 2017; Shen et al., 2020). This molecular cascade contributes to an increase in vascular permeability and extracellular matrix degradation in other tissues and likely does so in peripheral nerves as well (Deora et al., 2004; Phipps et al., 2011; Bharadwaj et al., 2013). These proposed effects of the re-localization of pANXA2 in injured nerves is thought to likely contribute to blood-nerve barrier breakdown and to recruitment of immune cells, namely, macrophages, in order to facilitate Wallerian degeneration (Dallacasagrande and Hajjar, 2020; Liu et al., 2019; Koerdt and Gerke, 2017; Sievers et al., 2003).

In uninjured peripheral nerve, annexin A2 is predominantly intracellular and enriched only in specific segregated areas such as Schwann cell cytoplasmic channels (Hayashi et al., 2007). Injury-associated signals such as calcium influx into axonal tissue and hydrogen peroxide release from the damaged axon trigger local Anxa2 mRNA translation and protein accumulation in Schwann cell pseudopods, consistent with injury-induced peripheral membrane targeting (Negro et al., 2018). Although explicit demonstration of Tyr23-phosphorylated annexin A2 on the Schwann cell outer surface *in vivo* is limited, it has been shown that calcium influx can drive phosphorylation-dependent translocation to the surface in neurons and chromaffin cells, supporting plausibility of a similar mechanism in injured peripheral nerve (Valapala et al., 2014; Grindheim et al., 2017; Gabel et al., 2019). Taken collectively, these dynamics make pANXA2 an attractive potential target for labeling injured nerve tissue.

LS-301 is a novel near-infrared (NIR) fluorescent probe that was originally developed for intraoperative tumor imaging capitalizing on its unique specificity for targeting pANXA2 and has already been approved for use in human trials in the oncological sphere (Shen et al., 2020). Chemically, LS-301 consists of a NIR fluorophore (heptamethine cyanine dye related to Cypate) conjugated to a cyclic octapeptide which was designed to bind with high affinity to the pANXA2 protein, but not its non-phosphorylated counterpart (Shen et al., 2020). In tumor models, LS-301 was shown to selectively accumulate in tumor regions where annexin A2 is phosphorylated and expressed on cell surfaces, such as invasive fronts of cancers and in tumor-associated macrophages and fibroblasts (Shen et al., 2020). Given this background, we theorized that the same molecular probe could be repurposed to label injured nerves and identify the presence of Wallerian degeneration, since the injury environment (elevated calcium, blood-nerve barrier disruption, Schwann cell activation) would likely mirror an increased presentation of pANXA2 targets on cell surfaces.

Given LS-301’s selective binding to pANXA2 and prior success in tumor imaging, we hypothesized that LS-301 would preferentially accumulate and demonstrate increased fluorescence in nerve segments undergoing Wallerian degeneration while showing minimal uptake in uninjured or recovering nerve tissue, thus providing high-contrast delineation of degenerating versus uninjured nerve tissue in a rat sciatic nerve injury model. The potential future clinical utility of this will be to evaluate neuromas in continuity and understand if the distal nerve has undergone complete Wallerian degeneration.

## Materials and methods

### Animal model

This study utilized an established rat model of sciatic nerve injury. Approximately 8-week-old adult wild-type Lewis rats (weighing approximately 200–250 g) were used for all experiments. All procedures were approved by the Washington University School of Medicine Institutional Animal Care and Use Committee (IACUC) and followed NIH guidelines for animal research. The rats were divided into experimental groups for each predetermined post-injury time point (with *n* = 8 rats per group per time point, roughly equivalent male to female ratio). In each animal, a unilateral sciatic nerve transection injury was performed, with the contralateral sciatic nerve serving as an internal control (sham surgery).

Specifically, under anesthesia using isoflurane gas, each animal’s sciatic nerve was exposed through a gluteal muscle-splitting incision. In the injury groups, the injury site was created by completely transecting (neurotmesis) the sciatic nerve with a sharp instrument at a standardized location approximately 1 cm distal to the sciatic notch which was first visualized within the proximal thigh, and the nerve ends were left unrepaired in order to model an acute injury scenario. On the opposite side from the injury, the sciatic nerve was similarly exposed to simulate identical operative conditions but was not transected in order to serve as the sham control for intra-animal comparison of probe uptake between injured and uninjured nerve segments as well as to be used for normalization in analysis for inter-animal comparisons across time points. The wound created in exposing the sciatic nerve with or without transection was closed in sequential layers consisting of muscle then skin, and animals were recovered from anesthesia after the initial surgery (Yi et al., 2017; Beirowski et al., 2005).

We evaluated nerve injury at three post-transection time points including 5 h, 2 days, and 2 weeks. These time points were chosen based on the expected timeline of Wallerian degeneration in a rat model (and clinically relevant time points) with 5 h representing the acute phase before substantial axon degeneration but when traumatic disruption of the blood-nerve barrier to impact probe uptake, 2 days representing early active degeneration, and 2 weeks representing a fully degenerated distal nerve (Yi et al., 2017; Lubińska, 1977).

### *In Vivo* imaging of fluorescent probes

Imaging probes (LS-301 and Cypate-3). LS-301 comprises a cypate-derived heptamethine cyanine dye (excitation ∼780 nm; emission ∼810–820 nm) covalently linked to a cyclic peptide with high affinity for phosphorylated annexin A2 (pANXA2). Cypate-3 is a spectrally distinct, untargeted near-infrared dye (excitation ∼680 nm; emission ∼700 nm) used as a control for non-specific *in vivo* uptake, including effects of injury-related blood-nerve barrier disruption. Probes were prepared per manufacturer instructions (PBS or DMSO/PBS), sterile-filtered, and dosed at 5 mg/kg each. Fluorescence imaging procedure.

Per the previously stated rationale, baseline images were acquired at 5 h, 2 days, and 2 weeks post-transection (time 0) prior to probe administration. This was followed by co-injection of both probes via a surgically implanted central venous catheter. LS-301 and Cypate-3 dyes were administered simultaneously and only once at 5 h prior to imaging irrespective of the post-injury time point being assessed. The 5 h prior to imaging were to allow the dyes the time to disseminate systemically based on prior experience with the probes. Following injection, animals remained under anesthesia on a warming pad for both the circulation duration and imaging duration. Images were acquired immediately after injection and at 5 h post-injection. Interim imaging was also done at 1 h post injection to ensure technically appropriate dye function and administration. Quantitative analyses used the 5 h post-injection images based on prior pharmacokinetic optimization.

Imaging was performed using a Pearl Impulse system (LI-COR Biosciences, Lincoln, NE) in the 800 nm channel for LS-301 and the 700 nm channel for Cypate-3, with brightfield acquired in parallel. Exposure settings were held constant across all scans to support intra- and inter-animal comparisons.

### Near infrared imaging analysis

Near-infrared fluorescence images were analyzed using LI-COR Image Studio software and ImageJ (NIH). Standardized regions of interest (ROIs) were placed over defined nerve segments: proximal bulb (PB; 3 mm proximal to transection), distal bulb (DB; 3 mm distal to transection), proximal adjacent (PA), distal adjacent (DA) (each 3 mm), and a sham ROI (10 mm) over the contralateral exposed sciatic nerve. Fixed-dimension ROIs were used across animals, and mean fluorescence was extracted for LS-301 and Cypate-3 in their respective channels after subtraction of background signal measured from a blank region outside the animal.

### Tissue collection and processing

Following *in vivo* imaging, rats were euthanized, and sciatic nerve stumps were collected at 5 h (n = 3), 2 days (n = 2), and 2 weeks (n = 3) post-injury. Tissues were embedded in OCT compound (Tissue-Tek®, Sakura Finetek Inc., Torrance, CA, United States), snap-frozen in liquid nitrogen, and sectioned coronally at 10 μm thickness using a cryostat. Sections were mounted on Superfrost™ Plus slides (Thermo Fisher Scientific, Waltham, MA, United States) and stored at −80 °C until further use.

### Immunofluorescence staining

Sections were fixed in either 4% paraformaldehyde for 1 h at room temperature or 100% chilled methanol for 10 min, according to antibody requirements. The following antibodies were used: anti-neurofilament (NF) (ab4680, 1:200; Abcam) with nuclear counterstain DAPI. The anti-NF antibody was conjugated with Alexa Fluor® 555 anti-Chicken IgG (Ab 150170, 1:200, Abcam). Sections were incubated with primary antibodies overnight at 4 °C, washed in PBS, and incubated with secondary antibodies for 2 h at room temperature before mounting in antifade medium with DAPI (ProLong™ Gold, Thermo Fisher Scientific).

### Immunohistochemistry imaging and processing

For the quantitative image analysis, images were acquired on a laser-scanning confocal microscope at ×40 magnification with 1024 × 1024 pixel resolution using identical acquisition settings across samples. Neurofilament (NF) signal was quantified using a custom Python pipeline (scikit-image v0.19) to minimize observer bias. Integrated fluorescence density was normalized to tissue area to report NF density (A.U./mm^2^) using a calibration factor of 0.357 µm/pixel.

### Statistical analysis

Fluorescence intensity was summarized as mean ± standard deviation by probe, time point (5 h, 2 days, 2 weeks), and nerve segment (proximal bulb, distal bulb, proximal adjacent, distal adjacent, and sham). Injured versus sham comparisons were performed within animals using paired t-tests with Bonferroni correction. To account for inter-animal variability, injured segments were normalized to the contralateral sham (Segment/Sham ratios) and analyzed by two-way ANOVA (Segment × Time) with Tukey’s *post hoc* testing. A three-way ANOVA (Probe × Segment × Time) evaluated the influence of probe type, segment, and time.

For NF quantification, the primary outcome was NF density, defined as integrated fluorescence density within the segmented mask normalized to tissue area (A.U./mm^2^). High-intensity artifacts (e.g., tissue folds or aggregates) were excluded as outliers >80,000 A.U./mm^2^ prior to analysis. Filtered NF density values were compared across time points by one-way ANOVA with Tukey’s *post hoc* test. For all tests, p < 0.05 was considered statistically significant.

## Results

Overall, near-infrared (NIR) imaging showed stronger discrimination of degenerating nerve for LS-301 than for Cypate-3 across the evaluated time points. Paired comparisons of each injured segment to the contralateral sham control identified segment- and time-dependent differences, with larger and more consistent effects for LS-301 (Table 1).

**TABLE 1:** Average raw fluorescence intensity of *in vivo* images. Average fluorescence intensity of each ROI compared to internal control Sham. Note the temporally distinct uptake pattern for LS-301 compared to the non-specific Cypate-3 probe.

​		LS-301 (*800 nm*)			Cypate-3 (*700 nm*)		
Region of interest	Time from injury	Average fluorescence intensity	Standard deviation	P values (*Change from sham*)	Average fluorescence intensity	Standard deviation	P values (*Change from sham*)
Proximal adjacent	5h	0.02	0.01	0.8424	0.03	0.02	0.0878
	2d	0.04	0.03	0.2908	0.05	0.04	0.4946
	2weeks	0.05	0.03	0.00529**	0.03	0.02	0.0275*
Proximal bulb	5h	0.03	0.01	0.6259	0.04	0.03	0.0431*
	2d	0.06	0.04	0.0524	0.08	0.08	0.1641
	2weeks	0.05	0.03	0.0017**	0.03	0.02	0.0759
Distal bulb	5h	0.03	0.02	0.0330*	0.04	0.03	0.0068**
	2d	0.07	0.05	0.0061**	0.10	0.14	0.1228
	2weeks	0.07	0.04	0.0010**	0.03	0.01	0.0087**
Distal adjacent	5h	0.03	0.01	0.4089	0.03	0.03	0.0456*
	2d	0.06	0.03	0.0005***	0.08	0.10	0.0974
	2weeks	0.06	0.04	0.0051**	0.03	0.02	0.0026**

* p < 0.05.

** p < 0.01.

*** p < 0.001.

At 2 weeks post-injury, LS-301 fluorescence was higher than sham in proximal adjacent (p = 0.0053), proximal bulb (p = 0.0017), distal bulb (p = 0.0010), and distal adjacent (p = 0.0051) segments. In the distal bulb, LS-301 was also increased at 5 h (p = 0.033) and 2 days (p = 0.0061) (Table 1). In contrast, Cypate-3 showed isolated increases relative to sham in the distal bulb at 5 h (p = 0.0068) and 2 weeks (p = 0.0087), and in the distal adjacent segment at 5 h (p = 0.0456) and 2 weeks (p = 0.0026) (Table 1).

**FIGURE 1:**
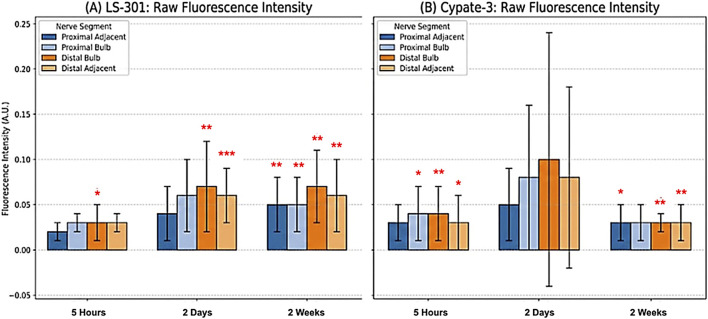
**(A,B)** Intra-time point analysis of raw fluorescence intensity. **(A)** demonstrates the raw fluorescence intensity of LS-301 for each ROI compared to the contralateral Sham control at 5 h, 2 days, and 2 weeks post-transection. LS-301 signal in the Distal Bulb (dark orange bars) was significantly elevated compared to the corresponding Sham segment at all time points. **(B)** illustrates the raw fluorescence intensity of Cypate-3 relative to Sham, demonstrates high variability in uptake without a discernible pattern of uptake (*) p < 0.05, (**) p < 0.01, (***) p < 0.001.

These patterns are visualized in Figure 1, with progressive LS-301 enrichment beginning in the distal bulb at 5 h and expanding by 2 days and 2 weeks (Figure 1A). Cypate-3 lacked a consistent spatiotemporal pattern and showed higher variability (Figure 1B). Representative *in vivo* images illustrate these differences (Figure 3).

**FIGURE 2:**
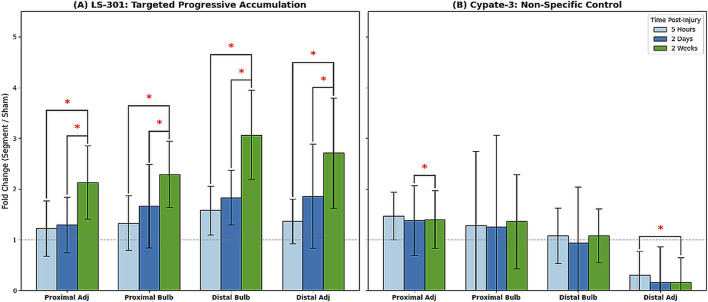
**(A,B)** Inter-time point comparison of normalized fluorescence intensities across time points. **(A)** shows the fold-change in LS-301 fluorescence intensity normalized to the internal Sham control. At 2 weeks the Distal Bulb exhibits the highest magnitude of uptake. **(B)** shows normalized Cypate-3 uptake, no specific temporal trend observed despite increased fluorescence compared to sham at multiple locations at 2 weeks. (*) p < 0.05.

LS-301 signal in uninjured sham nerves remained low at all time points, with no significant change over time (p > 0.05 for all comparisons), consistent with preferential uptake in injured nerve that was most pronounced at 2 weeks (Figure 2A). Cypate-3 did not exhibit a progressive increase in fluorescence intensity across the experimental timeline (Figures 1B, 2B).

To compare across time points, fluorescence was normalized to the contralateral sham nerve (Segment/Sham ratios; Table 2). LS-301 Segment/Sham ratios increased from 5 h to 2 weeks across segments, with peak accumulation in the distal bulb at 2 weeks (3.07 ± 0.88). The distal adjacent segment also increased between 5 h and 2 weeks (p = 0.001) (Table 2). A smaller increase in the proximal adjacent segment was consistent with the “dying back” phenomenon (Table 2; Figure 2A).

**TABLE 2:** A, B Normalized fluorescence ratios (Segment/Sham) of LS-301 and Cypate-3 Across Time Points. Segment/Sham ratios, normalized to internal contralateral controls (A). Ratios compared across time points (B). Data demonstrate significant progressive accumulation of LS-301 in all ROI segments of the injured nerve by 2 weeks.

A	Normalized fluorescence ratios				
Time point	Probe	PA: Sham	PB: Sham	DB: Sham	DA: Sham
5 h	LS-301	1.22 ± 0.55	1.33 ± 0.54	1.58 ± 0.48	1.36 ± 0.44
	Cypate-3	1.47 ± 0.47	1.28 ± 1.47	1.08 ± 0.55	0.30 ± 0.47
2 Days	LS-301	1.29 ± 0.55	1.66 ± 0.82	1.83 ± 0.54	1.86 ± 1.03
	Cypate-3	1.38 ± 0.69	1.26 ± 1.81	0.94 ± 1.10	0.16 ± 0.70
2 Weeks	LS-301	2.13 ± 0.72	2.29 ± 0.65	3.07 ± 0.88	2.71 ± 1.09
	Cypate-3	1.40 ± 0.57	1.36 ± 0.93	1.08 ± 0.53	0.16 ± 0.49

B	P values for inter-time point comparisons				
Comparison	Probe	PA: Sham	PB: Sham	DB: Sham	DA: Sham
5 hour vs. 2 Day (Acute vs. Early Degeneration)	LS-301	0.941	0.432	0.461	0.384
	Cypate-3	0.428	0.589	0.536	0.255
5 hour vs. 2 Week (Acute vs. Full Degeneration)	LS-301	0.004*	0.002*	<0.001*	0.001*
	Cypate-3	0.381	0.812	0.284	0.002*
2 Day vs. 2 Week (Early vs. Full Degeneration)	LS-301	0.009*	0.027*	0.002*	0.012*
	Cypate-3	0.015*	0.694	0.825	0.317

* p < 0.05.

Abbreviations: PA, proximal adjacent; PB, proximal bulb; DB, distal bulb; DA, distal adjacent.

Cypate-3 showed higher raw fluorescence than sham in the distal bulb at 5 h and 2 weeks (Table 1), but the distal bulb Segment/Sham ratio did not differ across time points (Table 2; Figure 2B). The only significant change in Cypate-3 Segment/Sham ratios occurred in the distal adjacent segment between 5 h and 2 weeks (p = 0.002) (Table 2; Figure 2B).

Two-way ANOVA (Segment × Time) for LS-301 showed significant main effects of segment (p = 0.035) and time (p < 0.001) without a segment × time interaction (p = 0.86), indicating spatial and temporal variation with a stable segmental pattern over time (Figure 2A). Cypate-3 showed a flatter, less specific profile (Figure 2B).

**FIGURE 3:**
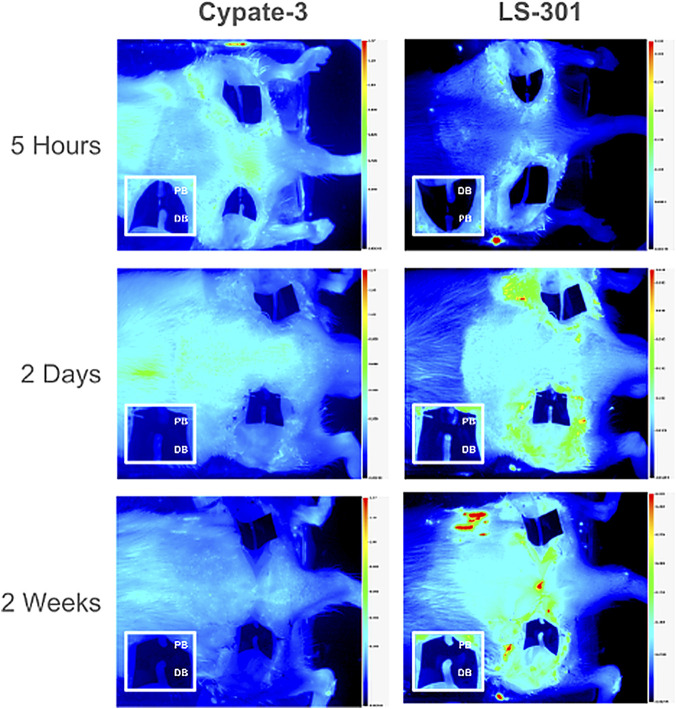
Distinct patterns of accumulation for p-ANXA2-specific LS-301 versus nonspecific Cypate-3 in distal nerve stumps. *In vivo* images demonstrate the sham and transected segments at 5 h, 2 days, and 2 weeks post-injury. Insets emphasize the distal and proximal nerve stumps. (Left Column) Cypate-3 shows non-specific uptake. (Right Column) LS-301 uptake pattern is consistent with the natural progression of Wallerian degeneration. Abbreviations: PB, Proximal Bulb; DB, Distal Bulb.

Three-way ANOVA (Probe × Segment × Time) similarly showed no segment × time interaction and identified significant effects of segment (p = 0.03) and time (p < 0.001). Probe type did not significantly affect total fluorescence uptake (p = 0.55), and probe × segment was not significant (p = 0.42). Probe × time was significant (p = 0.00078), consistent with distinct temporal uptake dynamics between LS-301 and Cypate-3 (Figure 3).

### Immunohistochemistry results

**FIGURE 4:**
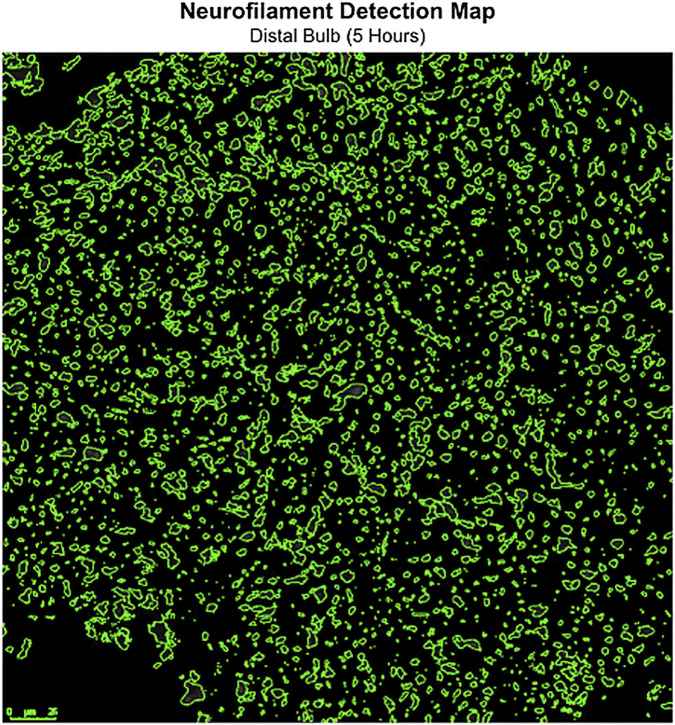
Visualization of neurofilament detection algorithm in the distal nerve. An example of the filament detection maps created and used in measuring the NF densities as surrogates to assess for axonal breakdown. Scale bar = 25 µm.

**FIGURE 5:**
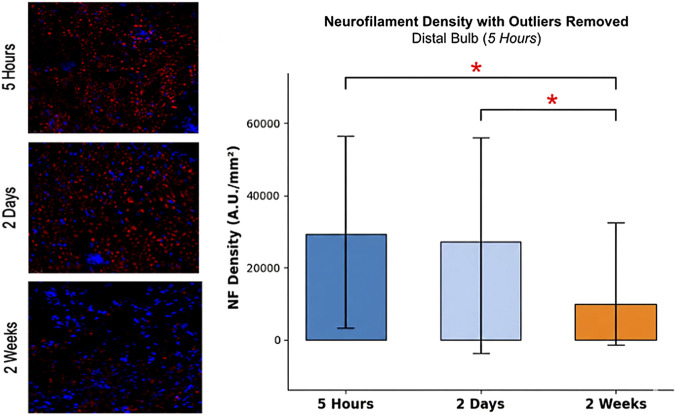
Imaging and quantification of neurofilament density. Quantitative analysis of neurofilament (NF) integrated density (A.U./mm^2^) in the distal bulb following artifact exclusion. Representative images display DAPI-stained nuclei (blue) and neurofilament (red) at each time point. Results indicate a significant decrease in NF density at 2 Weeks compared to both earlier time points (5 h and 2 days). (*) p < 0.05.

To confirm expected axonal degeneration in this model, distal bulb tissue from a subset of rats at each time point was stained for neurofilament (NF) and quantified (Figure 4). Figure 4 summarizes the automated quantification workflow used to determine NF density. After exclusion of staining artifacts using an outlier threshold, mean NF density was 29,278 ± 24,929 A.U./mm^2^ at 5 h and 27,113 ± 30,930 A.U./mm^2^ at 2 days (Table 3; Figure 5), with no difference by one-way ANOVA (p > 0.05). By 2 weeks, mean NF density decreased to 9,990 ± 19,835 A.U./mm^2^ (p < 0.05 vs. both 5 h and 2 days), consistent with the expected progression of Wallerian degeneration (Table 3; Figure 5).

**TABLE 3:** Summary Statistics of neurofilament density. Quantitative analysis of neurofilament (NF) density after the exclusion of staining artifacts (>80,000\A.U./mm^2^). Notable for a significant decrease in NF density at 2 Weeks compared to 2 Day and 5 h time points.

Time point	Mean	Standard deviation	N (Number of Images)	P values (Change from 2 Weeks)
5 hours	29,278	24,929	13	<0.05 *
2 Days	27,113	30,930	13	<0.05 *
2 Weeks	9,990	19,835	18	--

* p < 0.05.

## Discussion

In this study, we explored the use of LS-301, a phosphorylated annexin A2-targeted near-infrared fluorophore, as an indicator of Wallerian degeneration in a rat model of sciatic nerve transection. LS-301 uptake correlated strongly with the presence of Wallerian degeneration as indicated by preferential accumulation of the probe in nerve segments distal to the site of transection. Internal consistency with canonical Wallerian-degeneration timeline was demonstrated by NF quantification with IHC faithfully mirroring expected changes.

During the acute phase of Wallerian degeneration (0–48 h), the axonal cytoskeleton, represented by neurofilament density and organization, disassembles slowly with minimal loss of NF signal within the first 1–2 days (Gaudet et al., 2011). In analysis of NF density on IHC, there was no significant drop from 5 h to 2 days (p = 0.98) which aligns with the expected stability of neurofilaments in the distal bulb within the acute phase. Over days 3–14, Schwann cell and macrophage‐mediated clearance of cytoskeleton components begins to peak around days 3–5, with near‐complete axonal breakdown and removal by 14 days (Gaudet et al., 2011). This expected pattern was also demonstrated through IHC analysis that showed significant reduction at 2 weeks vs. 5 h (p = 0.035) indicating substantial NF breakdown by the end of this late phase further validating the presence of Wallerian degeneration occurring as expected within the context of this experimental model.

An increased NIR signal from the accumulation of LS-301 is seen primarily distal to the injury and in a portion of the proximal segment (representing the “dying back” phenomenon of Wallerian degeneration), while sparing truly uninjured tissue such as the contralateral sham control. As referenced, the pattern of LS-301 uptake became more distinct as the degeneration process progressed with increasing time from injury. The pattern was initially detectable in the acute phase (5 h and 2 days) but peaked most significantly at 2 weeks which aligns with the biological timeline of axonal degeneration and clearance seen with canonical WD. The non-targeted control dye, Cypate-3, by contrast, showed no such progressive spatiotemporal pattern which supports the notion that LS-301s behavior in uptake timing and intensity is not due to a generic property of NIR dyes or disruption of the blood-nerve barrier by injury alone both of which considerations are shared by the two probes. Rather these findings suggest strongly that LS-301s behavior is more likely due to its affinity for phosphorylated Annexin A2 and reflects the biological phenomenon of pANXA2 upregulation in nerve segments undergoing WD as occurs following transection injury. Thus, the LS-301 signal does not merely label the distal stump but, rather, dynamically delineates Wallerian degeneration compared to retained axonal architecture.

Taken collectively, these results hint at a possible mechanism by which LS-301 may be labelling injured nerves. Following transection, the blood-nerve barrier becomes more permeable initially due to mechanical disruption and subsequently due to molecular and cellular changes in the hours and days that follow​. This allows intravenously administered molecules, which would normally be excluded from the neural environment, to enter the endoneurial space of the transected nerve with an aim being to demonstrate that this is also the case for lesser injuries such as crush injuries with future data. This transection model supports LS-301s ability to bind to pANXA2, which is translocated to the surface of Schwann cells as the injury response of Wallerian degeneration unfolds (Shen et al., 2020). By 2 days post-injury, Schwann cells in the distal segment have dedifferentiated from their myelinating subtype and into a pro-regenerative subtype that is characterized by reactivity, proliferation state and the active digestion of myelin (Nocera and Jacob, 2020; Gomez-Sanchez et al., 2017; Kim et al., 2013; Höke et al., 2006). Studies have shown annexin A2 is upregulated in Schwann cells under injury or stress conditions (Negro et al., 2018). It is plausible that ANXA2 gets phosphorylated (at the previously demonstrated location of Tyr23) during this activation, although the precise timing of pANXA2 appearance in peripheral nerves requires further investigation (Shen et al., 2020). Our results suggest, but do not prove, that by 2 days there is sufficient pANXA2 present to bind LS-301, sequestering it to the injured nerve tissue especially in the distal stump. The cyclic peptide component of LS-301 has been shown to have high affinity for the pANXA2 epitope (Shen et al., 2020). As such, once this specifically targeted probe enters the nerve, it will bind to any exposed pANXA2 on cell surfaces, with increased visualized fluorescence corresponding to increased binding. LS-301 may also be taken up into the surrounding extracellular matrix if pANXA2 is shed or secreted by cells in the area such as Schwann cells. Once bound to pANXA2 on the cell surface, LS-301 may be internalized or remain bound in the nerve tissue (Shen et al., 2020). Prior studies using LS-301 to target solid organ tumors showed that after initial binding on cell membranes, the probe is endocytosed and trafficked to lysosomes leading to prolonged retention in target cells (Shen et al., 2020).

In the nerve, we theorize that a similar phenomenon occurs with LS-301 taken up by activated Schwann cells or macrophages that display an upregulated expression, translocation and phosphorylation of ANXA2 in response to the fluctuating milieu of cytokines, cells, and molecules that define the post-injury environment (Liu et al., 2019; Zhang et al., 2020). As such, uninjured nerve that lacks significant pANXA2 binding sites remains largely free of LS-301 after the circulation phase. The 5-h time point after injury is potentially somewhat confounded by the impact of overt BNB disruption induced by the transection allowing for an increase in uptake of nonspecific Cypate-3.

Compared to existing nerve imaging modalities, most of which are limited by barriers of cost, access, and inefficiency, LS-301 offers several advantages as an intraoperative nerve imaging agent for assessment of viability intraoperatively. This probe provides molecular specificity by targeting pANXA2 which serves to label only those nerves undergoing Wallerian degeneration, potentially identifying the zone of injury within a nerve. Utilization of a non-specific NIR reporter for determination of tissue viability has been widely used, but this is the first report of a nerve injury specific NIR probe. The emission wavelength of 800 nm makes it compatible for use with existing intraoperative near infrared cameras. Importantly, LS-301 has also already passed a major hurdle to practical utilization in that it is already in clinical trials for fluorescence imaging, albeit for the identification of solid tumors (Shen et al., 2020). In fact, LS-301 has been used with intraoperative imaging systems for tumors​ and is currently utilized in ongoing trials (NCI-2023-06503, NCI-2025-00366, NCI-2024-00905) demonstrating its satisfactory safety profile and ready applicability to nerve surgery (Shen et al., 2020). LS-301’s signal correlates with Wallerian degeneration in a biologically and therapeutically meaningful way. At 2 weeks, the proximal segment, which undergoes milder retrograde degeneration (‘dying back’), had significantly less fluorescence than the nerve distal to the transection that is fully separated from the axon cell body and thus undergoing full WD. While the timeline for WD in rats which has been discussed is different from that of human peripheral nerve injury in which the acute phase before substantial axon degeneration occurs within the first 3–4 days post-injury, the early active degeneration phase is observed between 4 and 8 days, and WD is completed in most nerves by 8–14 days, we would expect uptake at the corresponding time points to mirror the patterns seen in the rat model as the biologic changes that lend themselves to LS-301 binding occur in both rat and human peripheral nerve transection environments (Eder et al., 2017; Yi et al., 2017; Lubińska, 1977).

Despite these promising results, certain limitations to this study should be noted. Annexin A2 is present in many tissues, and although the phosphorylated form is more specific to certain conditions, inflammation or trauma in adjacent tissues could, in theory, cause some uptake of LS-301 outside the nerve. If surrounding muscle is severely injured, it might also become permeable and upregulate and/or excrete pANXA2 and other proteins that may bind LS-301 as is a common consideration with other Cypate dyes and their known binding to albumin (Shen et al., 2020). Co-localization of a Schwann cell marker like S100 with pANXA2 remains a priority for validation of LS-301s specificity for the process of WD. Accurate fluorescent measurement may require isolation of the nerve with a dark background. Additionally, this study utilized a transected nerve injury model and did not examine the effect of crush or avulsion, common mechanisms in high energy traumas though this is an active area of research in a parallel protocol. There remains a need to establish threshold criteria for what constitutes “positive” LS-301 uptake indicating active WD, removing the need for uninjured, contralateral nerves to serve as the internal control. Notably, in terms of possible future clinical applications, WD is expected and perpetuated in the segment distal to the site of injury. As such, a potential use of LS-301 is to help identify the extent of Wallerian degeneration in the distal nerve in neuromas in continuity. Additionally, the time points chosen for this study reflect potential clinically relevant time points for acute, subacute and delayed nerve repair, but may not capture the optimal time points for pANXA2 expression.

Future studies should address the above limitations and further evaluate if LS-301 would be fit for clinical translation in the context of nerve injury. Possible next steps may include time of administration and dosing optimization to determine the minimum dose of LS-301 and minimum uptake time needed to reliably visualize nerve injury based on scalable metrics such as height and weight. Partial injury models such as stretch or crush injuries should also be studied to see if LS-301 signal correlates with the severity of injury and/or functional outcomes.

## Conclusion

Our results provide proof of concept for this paradigm of “molecular fluorescence vision” of nerve injury. To our knowledge, this study is the first to demonstrate that a targeted NIR fluorescent probe (LS-301) can preferentially label injured nerve segments *in vivo* in a spatiotemporally dependent pattern that mirrors canonical WD while sparing uninjured nerves from significant off-target labeling.

These proof-of-concept data provide a foundation to further investigation of clinically relevant per-nerve surgical decision thresholds. Future studies are needed to test whether LS-301 mapping can complement electrodiagnostic evaluation and intraoperative inspection, and to define clinically actionable fluorescence thresholds.

## Data Availability

The raw data supporting the conclusions of this article will be made available by the authors, without undue reservation.
